# The Role of IAA in Regulating Root Architecture of Sweetpotato (*Ipomoea batatas* [L.] Lam) in Response to Potassium Deficiency Stress

**DOI:** 10.3390/plants12091779

**Published:** 2023-04-26

**Authors:** Ming Liu, Qiangqiang Zhang, Rong Jin, Peng Zhao, Xiaoya Zhu, Jing Wang, Yongchao Yu, Zhonghou Tang

**Affiliations:** 1 Xuzhou Institute of Agricultural Sciences of Xuhuai District of Jiangsu Province, China/Key Laboratory of Sweet Potato Biology and Genetic Breeding, Ministry of Agriculture/National Agricultural Experimental Station for Soil Quality, Xuzhou 221000, China; liuming0506@163.com (M.L.); xhszhang2021@163.com (Q.Z.); jinrong_2012@126.com (R.J.); zhaopeng0217@163.com (P.Z.); zxy15256131797@126.com (X.Z.); wangjing429645671@163.com (J.W.); yychaomail@163.com (Y.Y.); 2 School of Life Sciences, Jiangsu Normal University, Xuzhou 221116, China

**Keywords:** sweetpotato, lateral roots, auxin, potassium deficiency, stress tolerance

## Abstract

Plants can adapt to the spatial heterogeneity of soil nutrients by changing the morphology and architecture of the root system. Here, we explored the role of auxin in the response of sweetpotato roots to potassium (K^+^) deficiency stress. Two sweetpotato cultivars, Xushu 32 (low-K-tolerant) and Ningzishu 1 (low-K-sensitive), were cultured in low K^+^ (0.1 mmol L^−1^, LK) and normal K^+^ (10 mmol L^−1^, CK) nutrient solutions. Compared with CK, LK reduced the dry mass, K^+^ content, and K^+^ accumulation in the two cultivars, but the losses of Xushu 32 were smaller than those of Ningzishu 1. LK also affected root growth, mainly impairing the length, surface area, forks number, and crossings number. However, Xushu 32 had significantly higher lateral root length, density, and surface area than Ningzishu 1, closely related to the roots’ higher indole-3-acetic acid (IAA) content. According to the qPCR results, Xushu 32 synthesized more IAA (via *IbYUC8* and *IbTAR2*) in leaves but transported and accumulated in roots through polar transport (via *IbPIN1*, *IbPIN3*, and *IbAUX1*). It was also associated with the upregulation of auxin signaling pathway genes (*IbIAA4* and *IbIAA8*) in roots. These results imply that IAA participates in the formation of lateral roots and the change in root architecture during the tolerance to low K^+^ stress of sweetpotato, thus improving the absorption of K^+^ and the formation of biomass.

## 1. Introduction

Sweetpotato (*Ipomoea batatas* [L.] Lam) is an important raw material for grain, feed, and industrial processing in the world, and its planting area and total output in China rank first in the world [1]. However, sweetpotato is a typical ‘potassium (K^+^)-favoring’ crop, and its growth, development, yield, and quality are seriously affected by K^+^ deficiency stress [2]. Therefore, improving the resistance to low K^+^ stress and K^+^ utilization rate has become an essential issue in sweetpotato production and scientific research. Since plants can adapt to the spatial heterogeneity of soil nutrients by changing the root architecture [3,4,5], studying the mechanism of sweetpotato roots’ response to K^+^ deficiency and improving the root architecture conducive to K^+^ acquisition can provide a theoretical basis for the optimization of low-K-tolerance cultivation and a variety of improvements for sweetpotato.

As an essential macroelement, K^+^ regulates many plant growth and development processes, such as maintaining cell osmotic pressure, controlling stomatal development and closure, regulating enzyme activity, and participating in photosynthesis [6,7]. When K^+^ becomes the limiting factor, plants can obtain more K^+^ through different mechanisms, among which the change in root architecture is critical to improving K^+^ absorption. For example, low K^+^ stress significantly reduces the length, surface area, volume, and lateral root number of rice roots [8], similar to *Arabidopsis thaliana* [4]. In addition, there are differences in K^+^ nutrient use efficiency among different genotypes of the same crop. For example, maize varieties with low K+ tolerance under low K^+^ stress can promote potassium absorption by increasing root length, volume, and surface area [9]. There are also interspecific differences in K^+^ uptake and efficient use efficiency of sweetpotato [10]. In previous studies, our team also screened out the different K^+^ efficiency genotypes of sweetpotato and conducted physiological studies on low K^+^ tolerance [11]. However, there need to be more systematic studies on the different responses of sweetpotato root architecture to low K^+^ stress.

Endogenous plant signals regulate root growth and development, in which auxin is vital in root establishment [12]. A study showed that exogenous auxin significantly promoted the formation of lateral roots, and *superroot1*, *rooty*, *alf1*, and other mutants that overproduced indole-3-acetic acid (IAA) had significantly increased the number of lateral roots [13]. Auxin transport to the root is also crucial for root development. For example, when exogenous auxin transport inhibitors are applied, the concentration of auxin in the root decreases, accompanied by a decrease in the number and length of lateral roots [14]. In addition, the auxin signal transduction pathway also plays a vital role in lateral root development. For example, auxin signaling in *Arabidopsis thaliana* regulates root cell wall synthesis and remodeling by regulating the activities of two receptor-like protein kinases, MUS and MUL, thus controlling the early development of lateral root primordia [15]. Lateral root development is a crucial determinant of root architecture. Lateral root length, root density, and surface area are all essential components of root architecture. The spatial distribution of lateral roots plays a decisive role in the availability of nutrients reaching the root surface through diffusion [16]. Especially for root crops such as sweetpotato, lateral roots may also affect the formation of storage roots by changing the structure of adventitious roots [17]. Therefore, we speculated that the root architecture of sweetpotato in the early growth stage may be mainly determined by lateral root development and status. This process should be closely related to auxin synthesis, transport, and signal transduction.

There are few studies on the involvement of auxin in plant root growth under low K^+^ stress. Ma et al. [8] showed that K^+^ deficiency could cause root dysplasia in rice. This process was accompanied by changes in many related genes affecting auxin distribution and transport, indicating that auxin regulated root response to low K^+^ stress. The KUP4 mutant *TRH1*, a member of the KUP/KT/HAK family of Arabidopsis thaliana K^+^ transporters, had fewer root hairs and showed a reduced function of the up–down polar transport of auxins [18,19]. K^+^ deficiency treatment significantly inhibited root length and lateral root formation of cotton (shortening of branch area), mainly because the endogenous free IAA content in the roots decreased by more than 50% [20]. In a tobacco study, it was found that auxin polar transport genes *PIN1*, *PIN1b*, *PIN4*, and *PIN9* were involved in regulating the development of primary lateral roots of tobacco under low K^+^ stress [21]. These studies indicated that plants could adjust root growth and development to adapt to a low K^+^ environment through changes in auxin transport and distribution, which provided an important reference for studying the response of sweetpotato roots to low K^+^ stress.

However, the root architecture of sweetpotato is distinctly different from that of most dicotyledonous plants, such as *Arabidopsis thaliana* [22]. The mechanism of lateral root growth response to low K^+^ and the extent to which this reaction is related to auxin remains unclear. In this study, we observed the root architecture changes in sweetpotato seedlings, especially lateral roots, of two varieties with different low-K-tolerance under 12 days of low K^+^ stress. We measured IAA concentrations in seedling leaves and roots. We also examined the expression levels of auxin synthesis, transport, and signaling proteins. The results suggested that the low-K-tolerant variety under low K^+^ affected the lateral root elongation by regulating auxin synthesis and transport, thereby increasing the K^+^ uptake and accumulation.

## 2. Results

### 2.1. Variation in Biomass under Different K^+^ Levels

From day 3, the root biomass, shoot biomass, and total biomass of Xushu 32 and Ningzishu 1 under low K^+^ (LK) treatment were lower than those in the control treatment (CK) (Table 1). On day 6, the root biomass of both cultivars under LK treatment was significantly lower than that of the CK treatment, but the shoot biomass was not significantly different from that of the CK. After 12 days of treatment, the differences in root biomass, shoot biomass, and total biomass reached significant levels between different K^+^ levels. The root biomass, shoot biomass, and total biomass of Ningzishu 1 were significantly decreased by 21.70%, 26.76%, and 24.85%, respectively, compared with that of CK, which was higher than those of Xushu 32 (11.49%, 17.73%, and 15.57% decline, respectively).

### 2.2. Variation in K^+^ Concentration and K^+^ Accumulation under Different K^+^ Levels

The K^+^ concentration and K^+^ accumulation in the root and shoot of the two varieties increased with the extension of time under CK treatment, while they decreased significantly under LK treatment (Table 2). With LK treatment from day 3 to day 12, the K^+^ concentration in the root of Ningzishu 1 rapidly decreased from 28.54% to 8.36%, and the K^+^ concentration in the shoot decreased from 14.87% to 6.29%. In contrast, the K^+^ concentration in the root of Xushu 32 decreased slowly from 9.78% to 6.24%, while the K^+^ concentration in shoot decreased from 20.65% to 11.98%. In terms of K^+^ accumulation, that in root of Xushu 32 was significantly lower than that of Ningzishu 1 under LK treatment, but the K^+^ accumulation in the shoot was significantly higher than that of Ningzishu 1. After 12 days of LK treatment, the total K^+^ accumulation in the whole plant of Xushu 32 was 29.85 mg, 1.21 times higher than that of Ningzishu 1 (13.53 mg), and most of it was transported to the shoot, indicating that Xushu 32 had better absorption and transport capacity than Ningzishu 1 under LK treatment.

### 2.3. Variation in Root Architecture under Different K^+^ Levels

**Figure 1:**
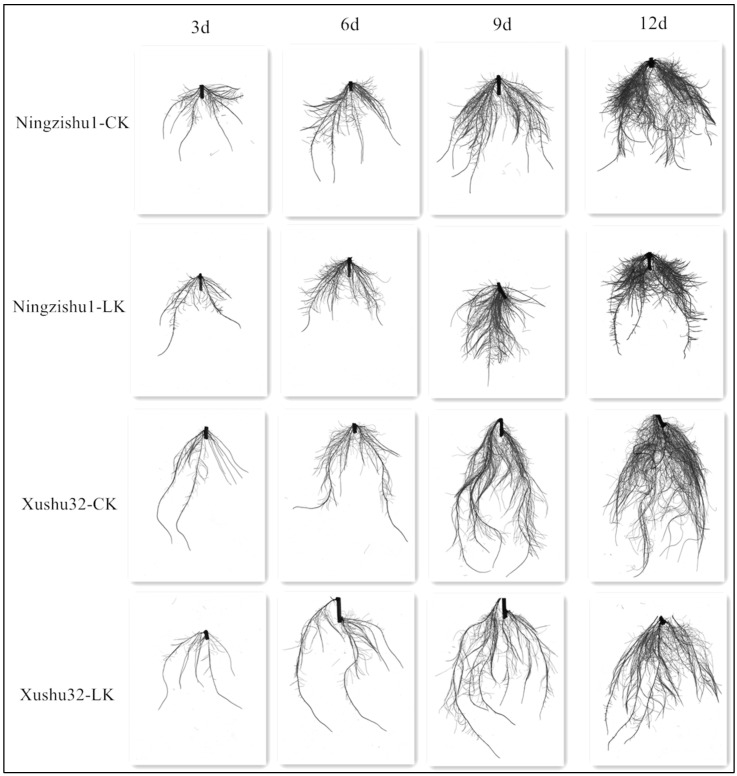
Root images of different sweetpotato varieties under different K^+^ treatments.

**Figure 2:**
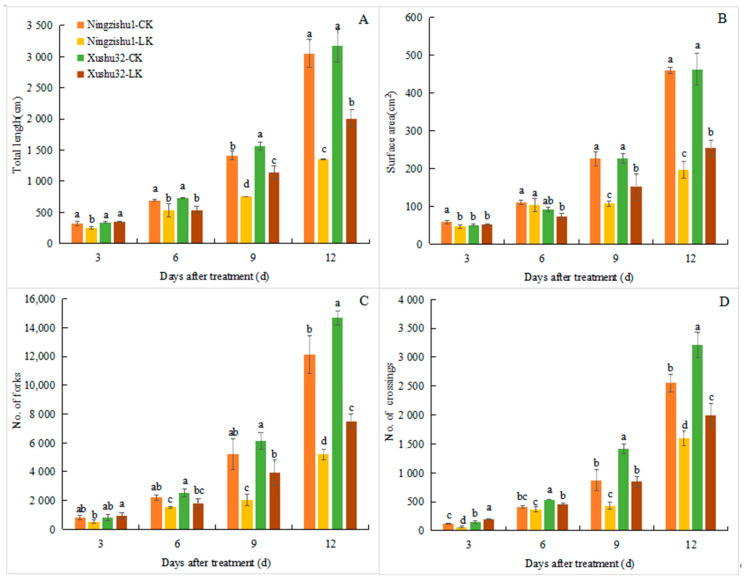
Root architecture of different sweetpotato varieties under different K^+^ treatments. (**A**) Total root length; (**B**) roots surface area; (**C**) no. of forks; (**D**) no. of crossings. Data are means ± standard deviation (*n* = 3); different letters (a, b, and c, etc.) on columns indicate significant differences between treatments on the same day (*p* < 0.05, Duncan’s test).

The root scanning images of different sweetpotato varieties under different K^+^ treatments are shown in Figure 1. Under LK stress, the root total length and surface area of Xushu 32 and Ningzishu 1 showed a downward trend from day 3 and were significantly lower than that of CK treatment on day 9 and day 12 (Figure 2). The root total length and surface area of Xushu 32 decreased by 36.85% and 45.17% on the 12th day, respectively, which were lower than those of Ningzishu 1 (59.94% and 57.07% decline, respectively). The number of root forks and crossings of the two cultivars were also suppressed by LK stress, and significant differences were detected from the sixth day (crossings number of Ningzishu 1 was from the ninth day). On day 12 of LK treatment, the number of root forks and crossings of Xushu 32 were significantly higher than that of Ningzishu 1, as well as the root total length and surface area.

**Figure 3:**
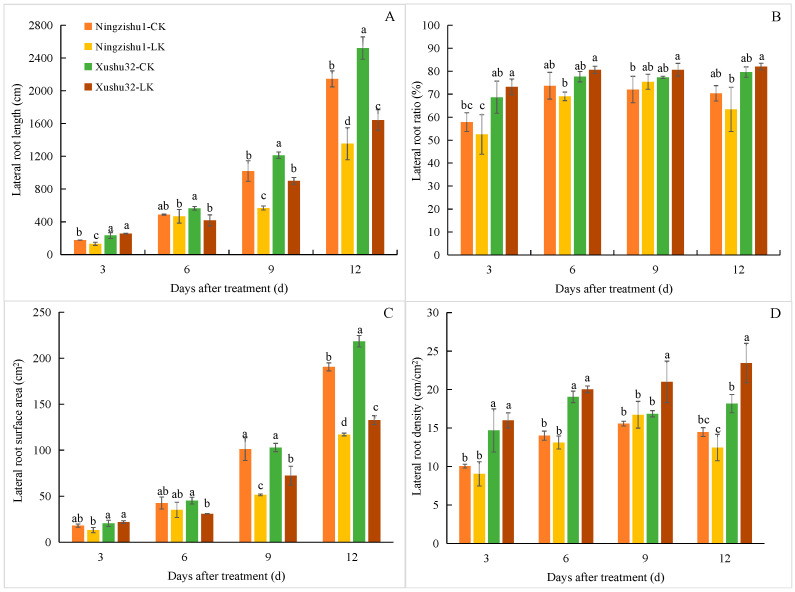
Lateral root architecture of different sweetpotato varieties under different K^+^ treatments. (**A**) Lateral root length; (**B**) lateral root ratio (=lateral root length/total root length × 100%); (**C**) lateral root surface area; (**D**) lateral root density (=lateral root length/total root projected area). Data are means ± standard deviation (*n* = 3); different letters (a, b, and c, etc.) on columns indicate significant differences between treatments on the same day (*p* < 0.05, Duncan’s test).

The length and surface area of lateral roots under LK treatment were also reduced compared with CK (Figure 3). However, the lateral root and surface area of Xushu 32 under LK treatment were significantly higher than those of Ningzishu 1 from day 6 to day 12. Until day 12 of LK treatment, the lateral root length and surface area of Xushu 32 were significantly higher than those of Ningzishu 1 by 21.39% and 13.47%, respectively. Nevertheless, compared with the CK treatment, LK treatment increased the lateral root ratio and density of Xushu 32, and the lateral root density significantly increased by 24.5% and 29.0% on the 9th and 12th day, respectively. In contrast, the ratio and density of lateral roots of Ningzishu 1 decreased under LK treatment (except on day 9); although these decreases were not significant compared with CK, they were significantly lower than those of Xushu 32.

### 2.4. Variation in IAA Content under Different K^+^ Levels

**Figure 4:**
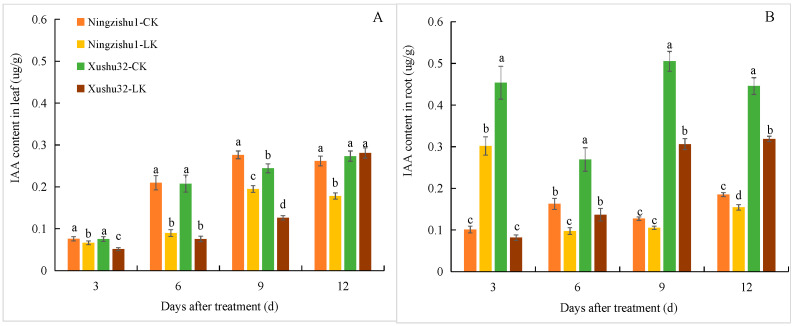
IAA content in leaf (**A**) and root (**B**) of different sweetpotato varieties under different K^+^ treatments. Data are means ± standard deviation (*n* = 3); different letters (a, b, and c, etc.) on columns indicate significant differences between treatments on the same day (*p* < 0.05, Duncan’s test).

The IAA content in Xushu 32 leaves continuously increased with the prolongation of LK treatment time, and in Ningzishu 1 leaves, the IAA content first increased but then decreased (Figure 4). The IAA contents in the leaves of Xushu 32 were lower than those in Ningzishu1 from day 3 to day 9 under LK, but, at 12 days, the IAA contents in Xushu 32 were significantly higher (57.86%) than those in Ningzishu 1. Under LK treatment, the IAA content in the roots of Xushu 32 roots also increased, but it first decreased and then increased in Ningzishu 1 roots, and was significantly lower than that of Xushu 32 at the 6th, 9th, and 12th days (by 28.5%, 65.6% and 106%, respectively).

### 2.5. Variation in Gene Relative Expression under Different K^+^ Levels

#### 2.5.1. Relative Expression of IbYUC8 and IbTAR2

**Figure 5:**
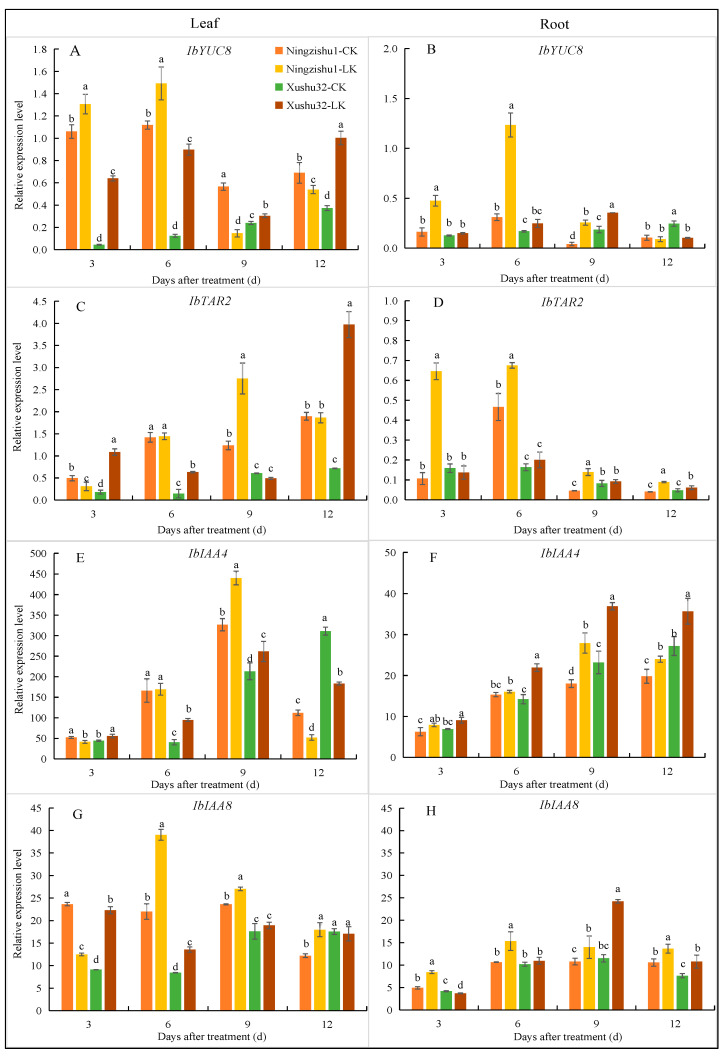
Relative expression levels of *IbYUC8* (**A**,**B**), *IbTAR2* (**C**,**D**), *IbIAA4* (**E**,**F**), and *IbIAA8* (**G**,**H**) in leaves (**A**,**C**,**E**,**G**) and roots (**B**,**D**,**F**,**H**) of different sweetpotato varieties under different K^+^ treatments. Data are means ± standard deviation (*n* = 3); different letters (a, b, and c, etc.) on columns indicate significant differences between treatments on the same day (*p* < 0.05, Duncan’s test).

The relative expressions of *IbYUC8* and *IbTAR2* in the leaves of Xushu 32 treated with LK were significantly higher than those of the CK (except IbTAR2 at day 9), and 2.7 times and 5.5 times higher than those of the CK at day 12, respectively (Figure 5A–D). In contrast, the relative expression of *IbYUC8* in the leaves of Ningzishu 1 was significantly increased on days 3 and 6, but decreased significantly after day 9. Additionally, the relative expression of *IbTAR2* in the leaves of Ningzishu 1 was significantly higher than that of the CK only at day 9, but had no significant difference to CK at day 12. During the first nine days of LK treatment, the relative expression of *IbYUC8* in the roots of the two varieties increased compared with the CK, but the increase in Ningzishu 1 was higher and the difference was significant. However, it showed no significant difference (Ningzishu 1) or even decreased (Xushu 32) on day 12. Unlike in the leaves, the relative expression of *IbTAR2* in the roots of Ningzishu 1 under LK treatment was significantly higher than CK, while that in Xushu 32 was significantly higher than CK but only on the 12th day. These results indicated that the IAA synthesis in the leaves of Xushu 32 was higher than in Ningzishu 1, while the IAA synthesis in the roots was lower than in Ningzishu 1.

#### 2.5.2. Relative Expression of IbIAA4 and IbIAA8

On the sixth and ninth days of LK treatment, the relative expression levels of *IbIAA4* and *IbIAA8* (IAA signal-related genes) in the leaves and roots of the two varieties increased to different degrees compared with CK, and the expression levels of *IbIAA4* and *IbIAA8* in the leaves of Ningzishu 1 were significantly higher than those of Xushu32 (Figure 5E–H). However, the expression levels in roots were significantly lower than those of Xushu 32 (except *IbIAA8* on the sixth day). On day 12, the relative expression level of *IbIAA4* in the leaves of Xushu 32 and Ningzishu 1 was significantly decreased under LK treatment, but it was still significantly increased in roots. The relative expression level of *IbIAA4* in the leaves and roots of Xushu 32 was significantly higher than that of Ningzishu 1 during the whole experiment.

#### 2.5.3. Relative Expression of IbPIN1, IbPIN3, and IbAUX1

**Figure 6:**
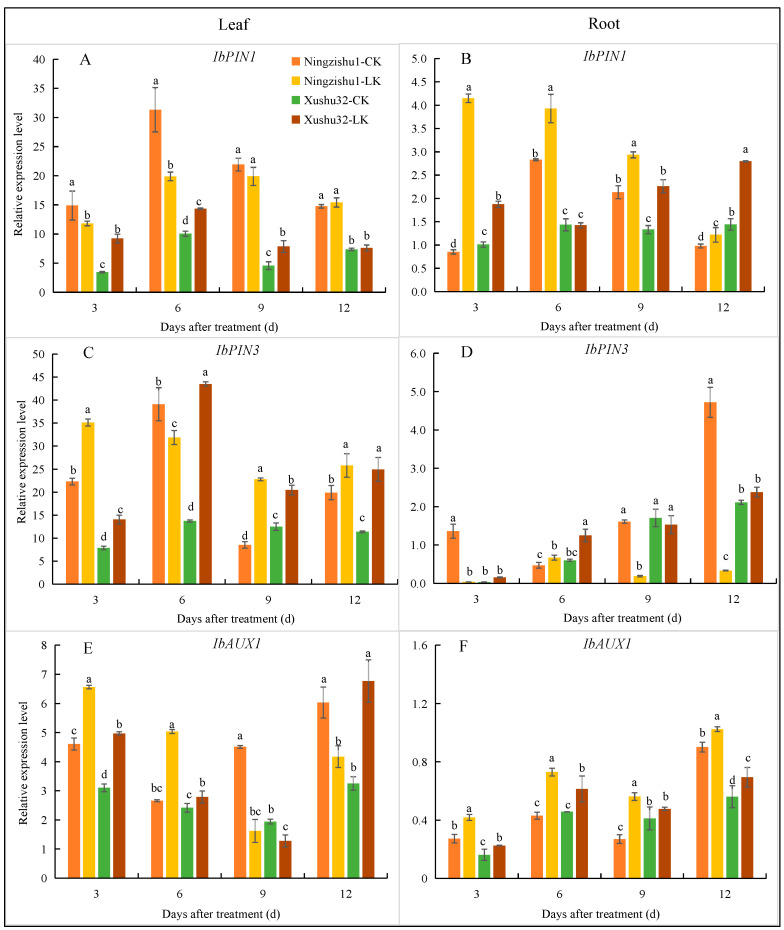
Relative expression levels of *IbPIN1* (**A**,**B**), *IbPIN3* (**C**,**D**), and *IbAUX1* (**E**,**F**) in leaves (**A**,**C**,**E**) and roots (**B**,**D**,**F**) of different sweetpotato varieties under different K^+^ treatments. Data are means ± standard deviation (*n* = 3); different letters (a, b, and c, etc.) on columns indicate significant differences between treatments on the same day (*p* < 0.05, Duncan’s test).

The results showed different expression patterns of *IbPIN1*, *IbPIN3*, and *IbAUX1* in different locations and varieties (Figure 6). Under LK treatment, the relative expression of *IbPIN1* in the leaves and roots of Xushu 32 was increased overall in the leaves and roots of Xushu 32, which was significant on days 3, 6, 9 in the leaves and 3, 9, 12 in the roots. In contrast, the relative expression of *IbPIN1* in the leaves of Ningzishu 1 under LK treatment decreased significantly in the first 6 days and there was no change in the last 6 days. The relative expression level of *IbPIN1* in the roots of Ningzishu 1 was significantly increased at first, but continuously decreased with the extension of the date, and was significantly lower than that of Xushu 32 on the 12th day. The relative expression of *IbPIN3* was significantly increased in the leaves of Xushu 32 under LK treatment, but only significantly increased in the roots on day 6. For Ningzishu 1, the relative expression level of *IbPIN3* in leaves increased significantly under LK treatment, but in roots decreased significantly except on the 6th day. Under LK treatment, the relative expression level of *IbAUX1* in the leaves and roots of Xushu 32 was significantly increased (except for on the 9th day), which was also significantly increased in the roots of Ningzishu 1, but it was significantly decreased in the leaves from the 9th day.

## 3. Discussion

K^+^ is the main osmotic cation in plant root cells, and K^+^ deficiency inhibits root growth, which has been demonstrated in many plants [4,8]. In *Arabidopsis thaliana*, two extreme strategies have been found for root adaptation to low K^+^ morphology: primary root growth is maintained and lateral root elongation is inhibited, or primary root elongation is blocked but lateral root branching is promoted [5]. At the early stage of sweetpotato growth, adventitious roots and their lateral roots develop into the water and nutrient absorbing root system [23]. However, it is not clear how sweetpotato adjusts root growth to cope with a low K^+^ environment.

In this study, K^+^ deficiency (LK) significantly reduced the uptake and accumulation of K^+^ in sweetpotato and inhibited the production of plant dry weight (Table 1 and Table 2), which was consistent with the results in rice and tobacco [21,24]. However, Xushu 32 (low-K-tolerant variety) showed less loss of biomass and potassium accumulation in the whole plant than Ningzishu 1 (low-K-sensitive variety), which indicated that Xushu 32 had certain adaptive strategies to deal with low K^+^ stress.

Compared with control treatment (CK), LK inhibited the root growth of sweetpotato plants mainly by affecting root elongation (Figure 2A) and branching (Figure 2C,D), and their changes were basically consistent with root surface area (Figure 2B). However, the lateral root length, proportion, and density of Xushu 32 were significantly higher than Ningzishu 1 under LK treatment, and the lateral root surface area was significantly different (Figure 3). This indicated that Xushu 32 promoted more branching and longer lateral roots in the LK environment, thus forming a root structure more conducive to K^+^ absorption, which was consistent with its increased accumulation of more K^+^ and biomass. Studies have shown that changing root morphology in response to K^+^ deficiency may help improve the nutrient efficiency and K^+^ deficiency tolerance of sugarcane [25], and rice roots with high efficiency genotypes can maintain a developed root structure to adapt to a low K^+^ medium [26]; these findings are similar to our research results. However, we can also see that although the root length and surface area of Xushu 32 under LK were higher than those of Ningishu 1, the root biomass (dry weight) was lower than that of Ningzishu 1. This may be due to the significant increase in lateral root ratio and density of Xushu 32 compared to Ningzishu 1, resulting in a significant decrease in root diameter (as shown in Figure S1, which was also significantly reduced under CK conditions). Moreover, there may be differences in the water content in the roots of these two varieties.

The growing roots continuously branch and form lateral roots to respond to different environmental conditions. In this process, auxin plays a leading role in initiating the founder cell of lateral root and in the later stage of lateral root development [27]. It has been confirmed that the decrease in auxin level is the main reason for the inhibition of the initiation and extension of tobacco lateral roots after K^+^ deficiency [21]. Our results showed that compared with CK plants, the IAA concentration in the leaves and roots of LK plants was significantly reduced (Figure 4), which should be directly related to the significant decrease in the length, surface area, forks number, and crossings number of roots under LK (Figure 2). However, there were differences in the tolerance of different varieties to LK, which was mainly manifested in that the length, surface area, and density of the lateral roots of Xushu 32 were significantly higher than those of Ningzishu 1 under the LK condition (Figure 3). This should be directly related to the higher IAA content in the roots of Xushu 32. However, IAA content in Xushu32 leaves was lower than Ningzishu 1 (except 12d) under the LK condition. This indicated that there were differences in the synthesis and distribution of IAA between the two varieties.

Environmental and endogenous signals may mediate changes in auxin distribution by influencing auxin synthesis and polar transport [28]. In *Arabidopsis*, auxin is synthesized mainly through a two-step dependent biosynthetic pathway catalyzed by the tryptophan aminotransferase (TAA) and flavin monooxygenase (YUCCA) enzyme families [29]. Through qPCR analysis of TAA and YUCCA family genes in sweetpotato, we found that *IbYUC8* and *IbTAR2* may be directly related to auxin synthesis. LK significantly up-regulated the relative expression levels of *IbYUC8* and *IbTAR2* in the leaves of Xushu 32. Still, the relative expression levels of *IbYUC8* and *IbTAR2* in the roots were not significantly up-regulated (except on day 9), and were much lower than those in the leaves (Figure 5). On the contrary, LK significantly up-regulated the relative expression levels of *IbYUC8* and *IbTAR2* in the roots of Ningzishu 1, but their changes in leaves were inconsistent. These results indicate that the IAA synthesis in Xushu 32 might be mainly in the leaves, while that of Ningzishu 1 might be primarily in the roots.

Auxin synthesized in most aboveground tissues is redistributed via auxin influx carriers such as AUX1/LAX family proteins and auxin efflux vectors including PIN and ABCB/PGP family proteins [30]. This study also showed that the polar transport genes *IbPIN1* (in leaves and roots), *IbPIN3* (mainly in leaves), and *IbAUX1* (in leaves and roots) (Figure 6) of Xushu 32 were significantly up-regulated by LK treatment. By comparison, the relative expressions of *IbPIN1* and *IbAUX1* in the roots of Ningzishu 1 also increased significantly, but they were decreased in leaves *(IbAUX1* was increased in early stage). In combination with the changes in IAA synthesis genes, we believe that the IAA synthesized in the leaves of Xushu 32 was transported to the roots by polar transport, which could also be the reason why the IAA content in Xushu 32 roots was significantly higher than that in Ningzishu 1. It has been shown that boron deficiency affects trifoliate orange root growth and development by affecting auxin synthesis gene expression *(TAA1*, *TAR2*, *YUC3*, and *YUC8*) and transporter gene expression (*AUX1*, *PIN1*, *PIN3*, *PIN4*, *LAX1*, and *ABCB1*) [31], which is similar to the results of this study. The Aux/IAA gene encodes a family of responsive proteins in the auxin signaling pathway, some of which (such as *OsIAA11* in rice) are involved in lateral root development [32]. In our experiment, the expressions of *IbIAA4* and *IbIAA8* in Xushu 32 roots were significantly up-regulated under LK, indicating that *IbIAA4* and *IbIAA8* were also involved in promoting lateral root development. However, the mechanism of synergy between these genes needs further study.

In addition to root morphology, research on the mechanism of low K^+^ tolerance in plants mainly focuses on K^+^ channel proteins (Shaker K^+^ channel family) and K^+^ transporters (KUP/HAK/KT potassium transporter family) in roots. In sweetpotato, it has been reported that *IbAKT1* [33] and *IbHKT-like* [34] may play important roles in the regulation of K^+^ deficiency tolerance in sweetpotato. Whether they have different roles in the two varieties in this experiment, regarding their interaction with root morphology and IAA, is worth further investigation.

## 4. Materials and Methods

### 4.1. Plant Materials and Growth Condition

The hydroponics experiment was conducted in the greenhouse of Xuzhou Institute of Agricultural Sciences in Xuhuai District, Jiangsu Province, China (34°27′ N, 117°29′ E) in the summer of 2021. Two cultivars with different tolerance to potassium (K^+^) deficiency stress (which had been verified in previous experiments [35,36], Xushu 32 (tolerant to K^+^ deficiency) and Ningzishu 1 (sensitive to K^+^ deficiency), were selected for this experiment. Healthy cuttings with four leaves, basal stem diameter of 12~13 mm, stem length of 20 ± 0.5 cm, and three internodes were selected from the seedling bed for cultivation in a hydroponic system. The hydroponic system mainly consisted of a 38 cm × 28 cm × 14 cm (H) plastic box with nutrient solution and a plastic lid with equidistant holes (7 cm apart). Each seedling was fixed in the planting basket with a sponge and put into the hole. Each pot was planted with 12 seedlings of the same variety. These cuttings were cultured in clean water for 3 days, and then added with the modified Hoagland nutrient solution (according to [35]). The nutrient solution was ventilated by an aeration pump.

### 4.2. Experimental Design and Sampling

Two K^+^ levels were set to 0.1 mmol L^−1^ (LK), and 10 mmol L^−1^ (CK, control) using K_2_SO_4_. The four treatments were labeled as Ningzishu 1 CK, Ningzishu 1 LK, Xushu 32 CK, and Xushu 32 LK, and six groups of replicates were set for each treatment. Plants were sampled on the day 3, 6, 9, and 12 after treatment. Three seedlings were randomly selected for each treatment, divided into roots and shoots, and dried in the oven to determine dry mass. The other three plants were separated into roots and leaves. The roots were first used for the scanning and analysis of root configuration, and then the roots and leaves were frozen in liquid nitrogen and stored in a −70 °C refrigerator for the determination of IAA content and relative gene expression.

### 4.3. Plant K^+^ Content and K^+^ Accumulation

After crushing the dried plant sample, we weighed 0.1 g and placed it in a 50 mL digestion tube. Then, 5 mL of concentrated sulfuric acid was added and heated on a digestion furnace to 350 °C for 2 h, and then the reaction liquid was catalyzed by 30% H_2_O_2_ until it was clarified. The K^+^ concentration of the diluted digestion solution was measured by flame spectrophotometry FP6410 (Shanghai Precision Instrument Co., Ltd., Shanghai, China) with the method of Gao et al. [37]. K^+^ accumulation was calculated by K^+^ content × dry weight.

### 4.4. Root Architecture Characteristics

EPSON V850 PRO Scanner (EPSON(China) Co., Ltd., Beijing, China) was used to scan the roots. After day 9, dense roots overlapped with each other (Figure 1), and they were divided into two or three parts for scanning and analysis. The WinRHIZO Root Analysis System (Regent Instruments Inc., Quebec, QC, Canada) was used to analyze the root architecture characteristics, such as root length, surface area, and number (NO.) of forks and crossings [35]. During the course of treatments in hydroponic culture, lateral roots growing on the adventitious roots developed obviously, and the diameter of lateral roots was mostly less than 0.5 mm. Therefore, roots with a diameter ≤ 0.5 mm were defined as lateral roots, and the length and surface area of lateral roots were also analyzed. Lateral root ratio was calculated by lateral root length/total root length × 100%; lateral root density was calculated by lateral root length/total root projected area.

### 4.5. Endogenous IAA Content

IAA was quantified and improved based on the method described by Fu et al. [38]. First, a certain amount of sample (0.5–0.9 g) was accurately weighed, anhydrous sodium sulfate was added in a ratio of 1:3.5, ground in a micromortar, and then transferred to a centrifuge tube. Next, 5 mL of petroleum ether was added, shaken well, and centrifuged for 3 min (3000 r/min). The upper ether phase was discarded. The remaining residue was extracted with 4.0, 2.0, and 2.0 mL of methanol in sequence according to the above method. The three extraction solutions were mixed and concentrated in a 10 mL conical scale tube to 2 mL, filtered through a 0.22 μM microporous membrane, and then 10 μL was taken out for sample analysis. The samples were analyzed by Liquid Chromatograph Mass Spectrometer AB SCIEX 4600 (LC-MS, Framingham, MA, USA) analysis. The chromatographic conditions were as follows: chromatographic column: HALO-C18 column (4.6 × 100 mm, 2.7 μm); mobile phase A: 0.5% formic acid aqueous solution; mobile phase B: 0.5% formic acid acetonitrile solution. Gradient elution was performed at the flow rate of 0.5 ml/min. The column temperature was 40 °C, and the detection wavelength was 272 nm.

### 4.6. Quantitative Real-Time RT-PCR Analysis

The relative expression analysis of the related genes was performed, and these were selected according to previous research [31,32,39]. An RNA extraction kit (DP432, TIANGEN) was used to isolate total RNA from the root and leaf. Total RNA was reversed transcribed into first-strand cDNA using PrimeScript RT Reagent Kit (code No. RR037A, Takara) and oligo-dT. We used SYBR Premium Ex TaqTM (Code: RR420A, Takara) to conduct qRT-PCR through the ABI 7300 sequencer according to the manufacturer’s protocol. The relative expression level of all genes was determined according to the 2^−△△CT^ method [40]. *Actin* was the reference gene. The primers used are listed in Table 3.

### 4.7. Statistical Analysis

The SPSS 20.0 program (SPSS Inc., Chicago, IL, USA) was used to statistically process the results. The data were expressed as mean ± standard deviation (*n* = 3). One-way ANOVA followed by Duncan’s multiple range test (*p* < 0.05) were used to determine the significant differences between treatments. The figures and tables were created using Microsoft Office 2016 (Beijing, China).

## 5. Conclusions

In conclusion, low K^+^ led to a decrease in dry mass, K^+^ content, and K^+^ accumulation of sweetpotato, thereby affecting root growth. In contrast, Xushu 32 suffered less loss, which may be related to its higher root length, surface area, forks number, and crossings number than Ningzishu 1. qPCR analysis showed that Xushu 32 synthesized more IAA (by *IbYUC8*, *IbTAR2*) in leaves and accumulated more IAA in roots through polar transport (by *IbPIN1*, *IbPIN3*, and *IbAUX1*). These changes in IAA contributed to the increase in the length, ratio, density, and surface area of the lateral roots of Xushu 32. These results suggest that auxin is involved in lateral root formation and root configuration changes in sweetpotato in response to low K^+^ stress.

## Figures and Tables

**Table 1:** Biomass of different sweetpotato varieties under different K^+^ treatments.

Days after Treatment	Treatment	Root Dry Mass (g/Plant)	Shoot Dry Mass (g/Plant)	Total Dry Mass (g/Plant)
3 d	Ningzishu 1-CK	0.16 ± 0.03 ^a 1^	1.00 ± 0.08 ^a^	1.16 ±0.08 ^a^
	Ningzishu 1-LK	0.13 ± 0.02 ^a^	0.90 ± 0.02 ^a^	1.03 ± 0.03 ^a^
	Xushu 32-CK	0.17 ± 0.03 ^a^	0.98 ± 0.03 ^a^	1.15 ± 0.09 ^a^
	Xushu 32-LK	0.15 ± 0.02 ^a^	0.88 ± 0.02 ^a^	1.04 ± 0.06 ^a^
6 d	Ningzishu 1-CK	0.21 ± 0.04 ^a^	1.37 ± 0.04 ^a^	1.58 ± 0.08 ^a^
	Ningzishu 1-LK	0.15 ± 0.03 ^b^	1.32 ± 0.03 ^ab^	1.47 ± 0.08 ^b^
	Xushu 32-CK	0.21 ± 0.02 ^a^	1.25 ± 0.03 ^bc^	1.45 ± 0.04 ^bc^
	Xushu 32-LK	0.15 ± 0.02 ^b^	1.20 ± 0.02 ^c^	1.35 ± 0.06 ^c^
9 d	Ningzishu 1-CK	0.27 ± 0.05 ^b^	1.71 ± 0.05 ^a^	1.98 ± 0.04 ^ab^
	Ningzishu 1-LK	0.17 ± 0.01 ^c^	1.61 ± 0.01 ^a^	1.78 ± 0.11 ^b^
	Xushu 32-CK	0.39 ± 0.04 ^a^	1.74 ± 0.04 ^a^	2.13 ± 0.09 ^a^
	Xushu 32-LK	0.33 ± 0.03 ^ab^	1.62 ± 0.03 ^a^	1.95 ± 0.10 ^ab^
12 d	Ningzishu 1-CK	1.29 ± 0.05 ^a^	2.13 ± 0.05 ^a^	3.42 ± 0.09 ^a^
	Ningzishu 1-LK	1.01 ± 0.03 ^b^	1.56 ± 0.03 ^b^	2.57 ± 0.17 ^c^
	Xushu 32-CK	0.87 ± 0.05 ^c^	2.03 ± 0.05 ^a^	2.89 ± 0.09 ^b^
	Xushu 32-LK	0.77 ± 0.05 ^d^	1.67 ± 0.05 ^b^	2.44 ± 0.08 ^c^

^1^ Data are means ± standard deviation (*n* = 3), means within the same column followed by different letters (^a^, ^b^ and ^c^, etc.) are significantly different between treatments on the same day (*p* < 0.05, Duncan’s test).

**Table 2:** K^+^ concentration and K^+^ accumulation of different sweetpotato varieties under different K^+^ treatments.

Days after Treatment	Treatment	K Concentration (%)		K Accumulation (mg/Plant)	
		Root	Shoot	Root	Shoot
3 d	Ningzishu 1-CK	3.77 ± 0.05 ^b 1^	1.72 ± 0.09 ^c^	6.15 ± 1.11 ^a^	17.13 ± 0.46 ^b^
	Ningzishu 1-LK	2.85 ± 0.06 ^c^	1.49 ± 0.12 ^d^	3.61 ± 0.60 ^b^	13.45 ± 1.51 ^c^
	Xushu 32-CK	4.44 ± 0.19 ^a^	3.26 ± 0.12 ^a^	7.59 ± 1.64 ^a^	31.78 ± 1.17 ^a^
	Xushu 32-LK	0.98 ± 0.02 ^d^	2.06 ± 0.07 ^b^	1.50 ± 0.14 ^c^	18.23 ± 1.10 ^b^
6 d	Ningzishu 1-CK	4.85 ± 0.02 ^b^	1.94 ± 0.06 ^b^	10.19 ± 1.79 ^a^	26.61 ± 2.15 ^b^
	Ningzishu 1-LK	1.92 ± 0.01 ^c^	1.19 ± 0.05 ^c^	2.88 ± 0.52 ^b^	15.75 ± 1.15 ^d^
	Xushu 32-CK	5.16 ± 0.10 ^a^	2.89 ± 0.15 ^a^	10.82 ± 0.72 ^a^	36.06 ± 2.30 ^a^
	Xushu 32-LK	0.98 ± 0.02 ^d^	1.90 ± 0.10 ^b^	1.51 ± 0.23 ^b^	22.74 ± 2.25 ^c^
9 d	Ningzishu 1-CK	5.79 ± 0.12 ^a^	1.61 ± 0.17 ^b^	15.68 ± 3.35 ^b^	27.56 ± 3.7 ^b^
	Ningzishu 1-LK	1.21 ± 0.06 ^c^	0.78 ± 0.09 ^c^	2.01 ± 0.07 ^c^	12.57 ± 0.87 ^c^
	Xushu 32-CK	5.63 ± 0.08 ^b^	2.25 ± 0.12 ^a^	22.15 ± 1.86 ^a^	39.34 ± 5.50 ^a^
	Xushu 32-LK	0.72 ± 0.06 ^d^	0.95 ± 0.03 ^c^	2.37 ± 0.37 ^c^	15.37 ± 1.14 ^c^
12 d	Ningzishu 1-CK	5.84 ± 0.40 ^a^	2.53 ± 0.10 ^b^	75.42 ± 7.87 ^a^	53.78 ± 1.31 ^b^
	Ningzishu 1-LK	0.83 ± 0.04 ^b^	0.63 ± 0.04 ^d^	8.39 ± 0.17 ^c^	9.84 ± 1.30 ^d^
	Xushu 32-CK	5.87 ± 0.05 ^a^	2.99 ± 0.08 ^a^	50.87 ± 2.49 ^b^	60.65 ± 2.04 ^a^
	Xushu 32-LK	0.67 ± 0.09 ^b^	1.20 ± 0.07 ^c^	5.14 ± 0.31 ^d^	20.01 ± 0.40 ^c^

^1^ Data are means ± standard deviation (*n* = 3), means within the same column followed by different letters (^a^, ^b^ and ^c^, etc.) are significantly different between treatments on the same day (*p* < 0.05, Duncan’s test).

**Table 3:** Sequence of primers for Actin and related Genes used for qRT-PCR.

Gene Name	Forward Primer	Reverse Primer
*Actin*	AGCAGCATGAAGATTAAGGTTGTAGCAC	TGGAAAATTAGAAGCACTTCCTGTGAAC
*IbYUC8*	ACCGCATGTTTAACTTCACCT	TCGAAGTGTTCAGCGTACGA
*IbTAR2*	GTACCGGAAGCTGTGTTTGC	TCCGCCATGCATCAGCTTAA
*IbPIN1*	CCCAGAAGCAAGCAGAGGAA	CACTTGGTTGGAAGCACAGC
*IbPIN3*	TGGCATGATCTTTACGTCGT	GGCGACAAAACGGTTGATCC
*IbAUX1*	CGCGTGGTATTTGGCCATTG	ATTGGTGGCTCCCGTAAAGT
*IbIAA4*	TGTGTTGCACATGAAGATGGTC	CACCATCACCTGTCTCTGCA
*IbIAA8*	CGAAATGTCTCCACCACTGC	CTCAGCTCAGTTGCCCTCAA

## Data Availability

The data presented in this study are available in the graphs and tables provided in the manuscript and Supplementary Materials.

## Supplementary Materials

The following supporting information can be downloaded at: https://www.mdpi.com/article/10.3390/plants12091779/s1, Figure S1: Root diameter of different sweetpotato varieties under different K^+^ treatments.
