# Supplementary figures and images for: The Role of IAA in Regulating Root Architecture of Sweetpotato (Ipomoea batatas [L.] Lam) in Response to Potassium Deficiency Stress
Source: Plants (Basel). 2023 Apr 26;12(9):1779. doi: 10.3390/plants12091779 (PMC10181447; doi:10.3390/plants12091779)

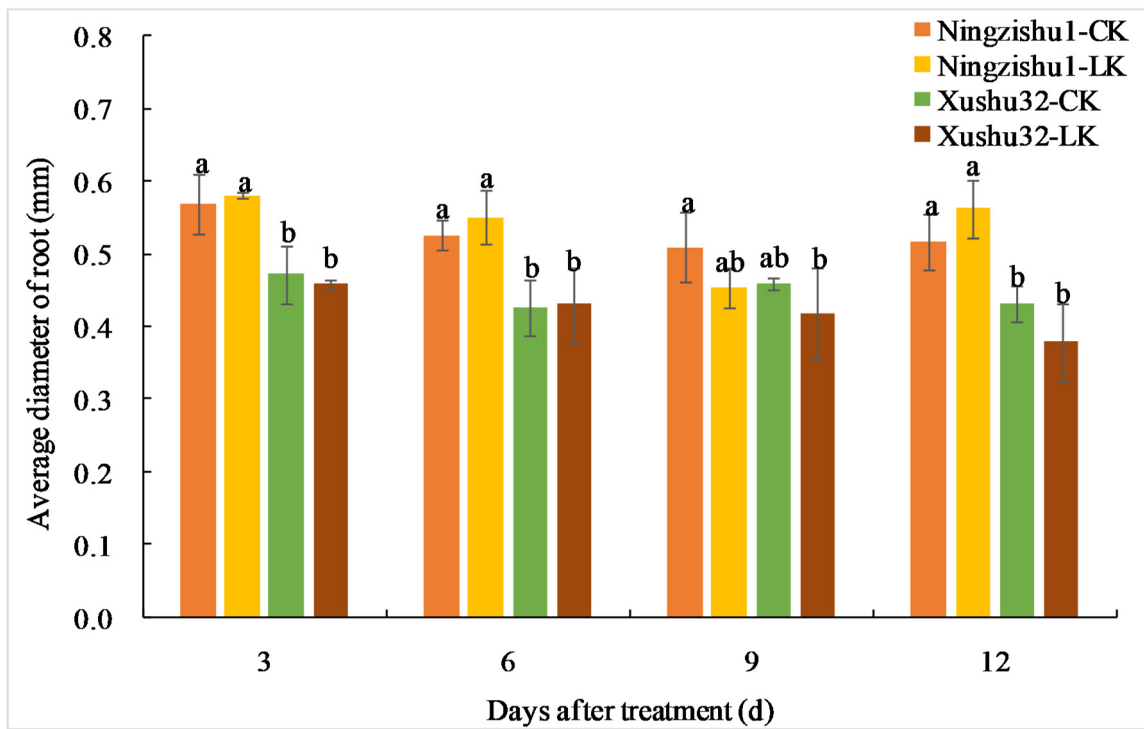

Figure S1. Root diameter of different sweetpotato varieties under different  $K^+$  treatments.

Supplement: Supplementary file 1 [file plants-12-01779-s001.zip › plants-2309772-supplementary.pdf]
